# Regulation of DEAH/RHA Helicases by G-Patch Proteins

**DOI:** 10.1155/2015/931857

**Published:** 2015-01-27

**Authors:** Julien Robert-Paganin, Stéphane Réty, Nicolas Leulliot

**Affiliations:** Laboratoire de Cristallographie et RMN Biologiques, UMR CNRS 8015, Faculté des Sciences Pharmaceutiques et Biologiques, Université Paris Descartes, Sorbonne Paris-Cité, 4 avenue de l'Observatoire, 75270 Paris Cedex 06, France

## Abstract

RNA helicases from the DEAH/RHA family are present in all the processes of RNA metabolism. The function of two helicases from this family, Prp2 and Prp43, is regulated by protein partners containing a G-patch domain. The G-patch is a glycine-rich domain discovered by sequence alignment, involved in protein-protein and protein-nucleic acid interaction. Although it has been shown to stimulate the helicase's enzymatic activities, the precise role of the G-patch domain remains unclear. The role of G-patch proteins in the regulation of Prp43 activity has been studied in the two biological processes in which it is involved: splicing and ribosome biogenesis. Depending on the pathway, the activity of Prp43 is modulated by different G-patch proteins. A particular feature of the structure of DEAH/RHA helicases revealed by the Prp43 structure is the OB-fold domain in C-terminal part. The OB-fold has been shown to be a platform responsible for the interaction with G-patch proteins and RNA. Though there is still no structural data on the G-patch domain, in the current model, the interaction between the helicase, the G-patch protein, and RNA leads to a cooperative binding of RNA and conformational changes of the helicase.

## 1. Introduction

Helicases have been historically defined as proteins able to unwind double-stranded (ds) nucleic acids in a nucleotide triphosphate- (NTP-) dependent manner [1, 2]. Sequence alignments revealed that overall sequence of helicases displays poor identity, but five superfamilies (SF) were defined from conserved motifs [3]. All these helicases possess RecA-like domains, so called because of the homology of this domain with* Escherichia coli* recombinase A [4, 5]. All the conserved motifs are located in the RecA-like domains and they constitute the structural and functional core of the helicase since it harbors NTP hydrolysis (Walker A and Walker B, Q motifs), nucleic acid binding, and helicase activities [6]. SF-1 and SF-2 helicases are monomeric but have tandem RecA-like domains, named RecA1 and RecA2, while SF-3, SF-4, SF-5, and SF-6 possess one RecA-like domain and are hexameric. In the case of SF-1 and SF-2, the two RecA-like domains are completed with ancillary regions in N-terminus and C-terminus or inserted in loops within the RecA-like domains. These regions modulate the activity or the specificity of the enzyme by autoinhibitory effects or by direct interaction with the nucleic acid and/or with a protein partner [7].

Helicases are often regulated by protein partners. In translation initiation, eIF4A is responsible for the unwinding of secondary structures during scanning of the 5′-UTR (5′-untranslated region). The activation and the recruitment of eIF4A need the interaction with eIF4B and eIF4G factors [8]. In ribosome biogenesis, the helicase Dbp8, involved in maturation of the small subunit, interacts with the nucleolar factor Esf2.* In vitro*, Esf2 can stimulate Dbp8 ATPase activity, suggesting that Esf2 also activates Dbp8 during ribosome biogenesis [9]. The RNA helicase activity of DEAH/RHA family helicases is also regulated by a special class of proteins that all contain G-patch domains (Table 1). The mechanisms of DEAH/RHA helicase activation by G-patch proteins are unclear and no structure of G-patch protein has been solved. In this review, we focus on the current knowledge about DEAH/RHA helicases regulation by G-patch proteins.

## 2. Functions of DEAH/RHA Helicases

The DEAH/RHA family (SF-2) is named after the sequence of the Walker B motif (Asp-Glu-Ala-His) and a member of the family, the RNA helicase A (RHA). The DEAH/RHA helicases are present in all essential processes of RNA metabolism such as transcription, translation, ribosome biogenesis, pre-mRNA splicing, or RNA sensing (Table 2). Members of the DEAH/RHA family are conserved across eukaryotes and contain the spliceosomal helicases Prp2, Prp16, Prp22, and Prp43 (resp., DHX16, DHX38, DHX8, and DHX15 in human) and of YLR419w (DHX29), Dhr1 (DHX37), and Dhr2 (DHX32). Other members of the family have no known homologues in yeast, such as DHX35, DHX9 (RNA helicase A), DHX57, DHX36, DHX30, DHX33, DHX40, and DHX34. Interestingly, many of these helicases are implicated in several different biological processes of RNA and/or DNA metabolism.

### 2.1. DEAH/RHA Helicases in Splicing

**Figure 1:**
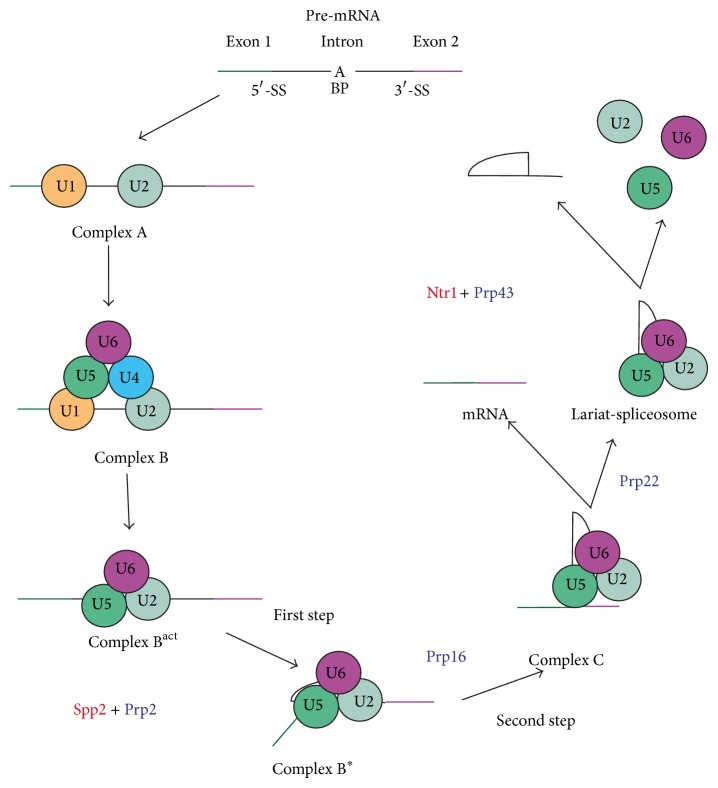
Schematic representation of the splicing cycle. Splicing is the removal of introns from pre-mRNA and ligation of exons in order to form the mature mRNA. The splicing cycle is a sequential set of reorganizations of the spliceosome, a complex composed of five snRNAs: U1, U2, U5, and U4/U6. U5 and U2/U6 constitute the catalytic core and catalyze the two nucleophilic attacks (catalytic steps). Four DEAH/RHA helicases (in black) and two known G-patch protein partners (in red) are involved in splicing. Prp2 is activated by Spp2 and acts prior to the first catalytic step. Prp16 acts between the two catalytic steps remodeling the spliceosome in order to permit the binding of essential factors for the second catalytic step. Prp2 is involved in the release of spliced mRNA from the lariat-spliceosome complex. Prp43 is activated by Ntr1 in the disassembly of the lariat-spliceosome complex. 5′-SS: 5′-splicing site, 3′-SS: 3′-splicing site, and BP: branch point.

The implication of DEAH/RHA helicases in pre-mRNA splicing has been intensively studied in yeast* Saccharomyces cerevisiae* [10]. DEAH/RHA helicases reorganize the different ribonucleoprotein (RNP) complexes during the splicing reaction and their mode of action is highly regulated because they all act at a precise step of the catalytic cycle. In pre-mRNA splicing, four DEAH/RHA helicases have been characterized in yeast (Figure 1). Spliceosomal remodeling by Prp2/DHX16 is responsible for the removal or displacement of the Bud13, Cwc24, Cwc27, and SF3a/b factors from the spliceosome prior to the first catalytic step and also creates binding sites for the Yju1 and Cwc25 factors [11]. Prp16/DHX38 is implicated in rearrangements of the spliceosome after the first catalytic step necessary for the second catalytic step and includes an indirect contribution to Cwc25 recycling [12]. Prp22/DHX8 is responsible for the release of spliced mRNA [13, 14]. Prp43/DHX15 catalyzes the disassembly of the lariat-spliceosome, recycling the components of the spliceosome and allowing degradation of the lariat [15, 16].

Interestingly, DEAH/RHA helicases are also responsible for proofreading of spliced pre-mRNA. Prp16 can discard aberrant spliceosomes which are stalled at the first catalytic step and Prp22 discards aberrant spliceosomes that are stalled in the second catalytic step. In the two cases, aberrant spliceosomes are disassembled by Prp43, indicative of cooperation between DEAH/RHA helicases in spliceosome proofreading. Spliceosomal DEAH/RHA helicases have been proposed to function as molecular clocks: an aberrant spliceosome is slower in its catalytic steps and Prp16 or Prp22 remodels them before the catalytic reactions can take place. According to these results, spliceosome proofreading is driven by a kinetic competition between RNP remodeling activity of DEAH/RHA helicases and catalytic steps of the spliceosome [17, 18].

### 2.2. DEAH/RHA in Ribosome Biogenesis

Prp43 is remarkable because this helicase is required in two distinct pathways: splicing and ribosome biogenesis [19–21]. Prp43 is implicated in the biogenesis of the two ribosomal subunits and binds several sites on the pre-rRNA during the biogenesis. In budding yeast, a lack of Prp43 results in accumulation of pre-rRNA intermediates from both subunits [19–21], showing that Prp43 is one of the only factors implicated in the biogenesis of the two ribosomal subunits. Prp43 coprecipitates with RNA polymerase I, indicating that it associates with preribosomal particle on the nascent pre-rRNA [19–21]. CRAC experiments identified several binding sites of Prp43 on the pre-rRNA. One major site is located at the helix 44 of the 20S, close to the processing site D, supporting previous results implicating Prp43 in the regulation of D site cleavage by endonuclease Nob1 [22]. In these experiments, Prp43 also was cross-linked with several box C/D snoRNA binding sites such as helix 39/40. The fact that Prp43 immunoprecipitates with several snoRNA, that a Prp43 mutant impedes methylation of 27S by a C/D box snoRNA, and that depletion of Prp43 trapped snoRNAs in the preribosome supports the model in which Prp43 functions to remove snoRNA from pre-rRNA [19–21, 23].

### 2.3. DEAH/RHA in Translation Initiation

The role of DEAH/RHA helicases in translation initiation has mostly been investigated for the human homologue of YLR419w (DHX29) and DHX9. The DHX29 helicase is essential in translation initiation during the formation of the 43S complex, composed of the eIF2/GTP/Met-tRNAi^Met^ complex, initiation factors, and the ribosomal 40S subunit. DHX29 favors scanning of the mRNA by the 40S subunit in presence of stable secondary structures and mediates base-pairing between initiation codon and tRNAi^Met^ in order to form stable 48S complex [24]. The cryoEM structure of the 43S complex bound to DHX29 brought new insights into the mode of action of this helicase. The location of DHX29 on the structure suggests that the helicase does not directly contact mRNA but suggests that it remodels the 40S subunit structure, thereby favoring mRNA secondary structure unwinding in an indirect manner [25]. DHX9 is another DEAH/RHA helicase implied in translation initiation. This helicase stimulates translation of mRNA containing a PCE (posttranscriptional control element) but its mode of action and precise role remain elusive [26].

### 2.4. Other Roles of DEAH/RHA Helicases

A role of metazoan DEAH/RHA helicases in viral RNA sensing and immune responses has been elucidated more recently. DHX33 and DHX9 are able to sense viral RNA and to activate IPS-1 mediated signalization in order to stimulate the production of inflammatory cytokines in myeloid dendritic cells [27, 28]. The RNA-sensing activity of DHX29 has been demonstrated in human airway system cells [29]. It acts together with the RIG-I helicase, a well-characterised sensor of immunity, and interacts with viral RNA and activates the RIG-I-MAVS pathway by its CARD domains. The direct interaction between MAVS, DHX9, RIG-I, and nucleic acid triggers MAVS signaling pathway. Altogether these results indicate that DHX9 would act as a cosensor of RIG-I [29]. DHX15 has been related to viral infection related apoptosis and cytokines production. In this context, DHX15 senses viral RNA and interacts directly with MAVS in order to trigger signaling by the NF-*κ*B pathway [30, 31].

In addition to their previously described functions, DHX9 and DHX33 have also been described in other processes. DHX9 was found to be essential for genomic stability. DHX9 is able to bind to intramolecular triplex DNA, hot-spots of mutations in human genome, and to prevent mutations and genomic instability, probably by acting on DNA structure [32]. The DHX33 helicase has been identified as a mediator of rRNA synthesis by promoting the access of RNA polymerase I to the rDNA loci. Its acts by remodeling rDNA structure and associating with the chromatin modulating protein UBF [33].

## 3. Regulation of DEAH-Box Activity by G-Patch Protein Partners

### 3.1. G-Patch Domain and G-Patch Proteins

**Figure 2:**
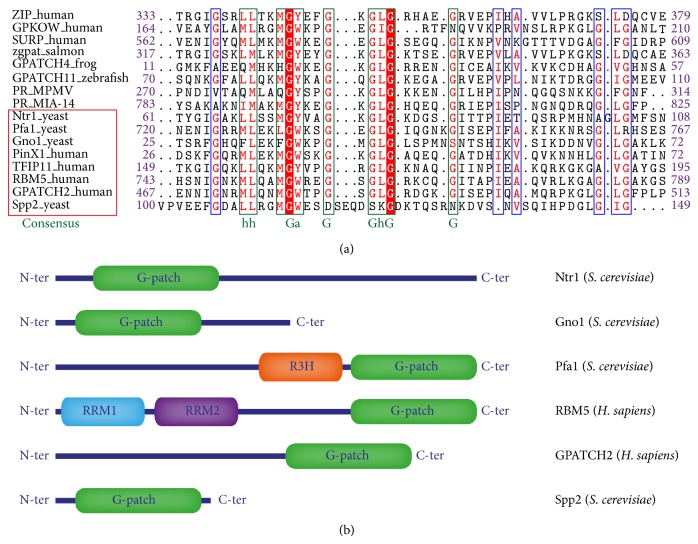
Sequence and organization of G-patch domains and G-patch protein partners. (a) Sequence alignments of G-patch domains from various G-patch proteins from different organisms. Sequences from G-patch protein partners of DEAH/RHA helicases from human and yeast are in the red box. The consensus sequence of G-patch is written in green: G is a glycine, a represents an aromatic residue, and h represents a hydrophobic residue. G-patch domain is a 50 AA glycine-rich domain with some residues invariably conserved like the aromatic residue after the first glycine. (b) Schematic representation of G-patch protein partners of DEAH/RHA helicases and their domains.

The G-patch domain was identified by sequence alignment as a 45–50 amino acids conserved motif with a consensus* hhx*(3)G*ax*(2)G*x*G*h*G*x*(4)G where *a* is an aromatic residue,* h* is a hydrophobic residue, and *x* is a number of positions occupied by nonconserved residues (Figure 2(a)) [34]. According to secondary structure predictions, the G-patch is composed of two *α*-helices flanked by loops. In this first study, G-patch domains were found in eukaryotic proteins that contained RNA-binding domains such as SWAP, RRM, or R3H. This association with RNA-binding domains and the fact that G-patch domains were also found in proteins involved in splicing or transport of mRNA led to the assumption that these domains are involved in protein-RNA interactions.

In proteinases and reverse transcriptase (RT) from betaretroviruses, the G-patch domain was shown to be both protein-nucleic acid and protein-protein interaction domain. Retroviral proteinases from Mason-Pfizer virus (MPMV) and mouse intracisternal A-type particles endogenous retrovirus (MIA-14) contain a G-patch domain in its C-terminal domain. Electrophoretic mobility shift assay demonstrated that the G-patch domain of these proteinases is responsible for the association with single-stranded nucleic acids (DNA and RNA) without sequence specificity [35]. In addition, the G-patch of proteinase of MPMV is important for infectivity but this function does not seem to be linked with the protease activity. The G-patch associates with the reverse transcriptase suggesting that it can function as a protein-protein interaction module [36, 37]. This interaction increases RT activity possibly by maintaining a favorable conformation of the substrate RNA. The G-patch domain also potentially mediates the interaction of the MPMV proteinase with breast cancer-associated protein BCA3 [38].

The transcription repressor protein ZIP contains a G-patch and is also involved in both protein-nucleic acid and protein-protein interactions. ZIP can repress the expression of the oncogene EGFR by the recruitment of the NuRD complex through its coiled-coil domain [39]. Interestingly, ZIP is expressed in another shorter isoform called sZIP that lacks the Zn finger, Tudor, and the first ten residues of the G-patch domains. This isoform is unable to bind DNA but interacts with the NuRD complex in competition with ZIP [40]. It is tempting to assume that the truncation of the G-patch domain coupled to the lack of Tudor and Zn finger domains is responsible for the loss of the interaction with DNA in ZIP. ZIP is able to dimerize and residues 361 to 430 are crucial for this dimerization. Interestingly, this region includes 18 residues of the G-patch domain [41].

G-patch domains are also found in several protein partners responsible for the activation of DEAH/RHA helicases. There are six known G-patch proteins interacting with DEAH/RHA helicases: RBM5, Ntr1/TFIP11, Gno1/PinX1, Spp2, Pfa1/Sqs1, and GPATCH2. Sequence alignments of G-patch domains and domains compositions of these proteins are represented in Figure 2(b). Alignments show that the aromatic residue after the first glycine is always conserved and is a tryptophan or a tyrosine. Glycine positions are well conserved except for the fifth glycine of Gno1 which is occupied by a serine. G-patch domain of Spp2 seems to be less conserved (Figure 2(a)). The G-patch is the only remarkable domain for GPATCH2, Spp2, Gno1, and Ntr1. However Pfa1 and RBM5 also display domains and motifs involved in RNA binding such as Zinc fingers, R3H, or RRMs (Figure 2(b)). As it was originally defined, the G-patch domain is associated with nucleic acids binding domains.

### 3.2. *In Vivo* Evidence of Regulation of DEAH-Box Helicases by G-Patch Proteins

The first activator of a DEAH/RHA helicase to be characterized is Spp2, a Prp2 regulator in splicing of pre-mRNA [42–45]. In budding yeast, Spp2 has been shown to interact with the spliceosome prior to the first catalytic step of splicing. Spp2 is required for efficient splicing and cell extracts depleted for Spp2 are blocked prior to the first catalytic step. Prp2 and Spp2 interact physically and the interaction is necessary for the activation of Prp2 function [42, 44].

The bifunctional Prp43 helicase is recruited and activated in splicing and ribosome biogenesis by different G-patch proteins. The function of Prp43 in splicing is mediated by the Ntr1 protein [46]. Ntr1 is a G-patch protein that interacts with the NineTeen related complex by the protein Ntr2 [47]. Splicing assays* in vitro* confirmed that Ntr1 was responsible for the activation of Prp43 in lariat-spliceosome disassembly [46]. Ntr1 forms a stable complex with Ntr2 and the Ntr1/Ntr2 complex associates with U5 by a direct interaction with Ntr2 [48]. Prp43 is recruited to the spliceosome by the Ntr1/Ntr2 complex, thereby targeting the helicase activity of Prp43 for spliceosome dissociation [47]. Interestingly, TFIP11, the human homolog of Ntr1, also possesses a G-patch domain and colocalizes with DHX15, the human homologue of Prp43 [49]. This interaction has been confirmed by isolation of postsplicing intron-lariat complexes where deletion of TFIP11 impairs spliceosome disassembly by DHX15 [50]. The G-patch protein RBM5 present only in metazoans has been shown to be a regulator of alternative splicing in apoptosis. Since RBM5 is able to activate helicase and ATPase activity of DHX15, it probably regulates splicing by activation of DHX15 [51].

The activity of Prp43 in ribosome biogenesis is stimulated by G-patch proteins Pfa1 and Gno1 (PinX1 in human) [19, 52]. Immunoprecipitation demonstrates that Prp43 associates with Pfa1 and pre-40S [19]. Other experiments have shown that Pfa1 is associated with the 90S, pre-40S, and pre-60S subunits [53]. Depletion experiments have demonstrated a genetic link between Pfa1 and Prp43 and the protein Ltv1 in ribosome biogenesis. Cells depleted for Ltv1 and lacking Pfa1 display an impairment of pre-rRNA processing. Complementation of these cells with Pfa1 and northern blot analysis of pre-rRNA show that Pfa1 plays a role in cleavages at sites A1 and A2 on 35S pre-rRNA and D cleavage site on 20S pre-RNA [53]. The stimulation of Prp43 function by Pfa1 in order to promote site D cleavage has been confirmed by the combination of* in vitro* and depletion experiments [22] and probably functions by promoting the release of snoRNA. No known homologues exist in humans for Pfa1, but G-patch proteins of unknown function that regulate DHX15 function, such as GPATCH2, may act as functional homologues in ribosome biogenesis [54].

Gno1 is important for pre-rRNA processing and maturation because deletion of this protein leads to accumulation of the 35S precursor [55]. Northern blot, immunoprecipitation, and pulse-chase analysis in ΔGno1 yeast strains show that Gno1 is recruited to the 90S and remains associated with the pre-60S and pre-40S. When Gno1 is deleted there is a severe accumulation of 20S and 27SB pre-rRNA in yeast.* In vitro* and* in vivo* data indicate that Gno1/PinX1 interacts with Prp43/DHX15 and probably triggers its function [52].

Interestingly, neither Pfa1 nor Gno1 is essential to the recruitment of Prp43 to the preribosome [19]. This is in contrast with G-patch proteins Spp2 and Ntr1 that are essential to the recruitment of Prp2 and Prp43 to the spliceosome. In ribosome biogenesis, the G-patch proteins only seem to activate Prp43 but do not recruit it. The fact that Prp43 is not specifically recruited to the preribosome can explain why several binding sites are detected by cross-link experiments [23]. The helicase could bind to several sites and need the interaction of Gno1 or Pfa1 in order to trigger its activity at specific sites.

Deletion of Gno1 reduces the accumulation of Pfa1 in preribosomal particle, although deletion of Pfa1 does not affect Gno1 levels [53]. Despite this result, no direct interaction between Gno1 or its human homologue PinX1 and Pfa1 has been detected in pull-down assays [52, 53]. However, human PinX1 and TFIP11 were shown to interact by two-hybrid experiment and copurification from bacterial expression system [56]. A functional link between these activators is therefore still to be demonstrated.

### 3.3. Mechanism of Activation of DEAH/RHA Helicases by G-Patch Proteins

In order to characterize the specific role of the G-patch domain in regulation of helicase activity, functional studies have focused on the interaction between G-patch proteins and helicases and how this interaction modulates helicase activity. The G-patch proteins are able to interact with Prp43* in vitro* and to form a stable complex. The N-terminal domain (residues 1–120) of Ntr1 containing the G-patch is sufficient to interact with the Prp43 helicase and mutations in conserved residues of the G-patch domain disrupt the interaction [46]. In human cells, RBM5 is able to interact with DHX15 [51]. Similar results have been obtained with Pfa1 [53]; the Pfa1 C-terminal domain (574–767) containing the G-patch and the Pfa1 N-terminal domain (1–202) are able to form complex with Prp43. Therefore, Pfa1 possesses two distinct binding sites with Prp43 and only one of these sites contains the G-patch domain [53]. The interaction between Gno1 and Prp43 has been demonstrated by coimmunoprecipitation in yeast. Interestingly, Prp43 is also able to interact with its human homologue PinX1 and mutations of conserved residues in the G-patch domain of Gno1 or PinX1 reduce the interaction with Prp43. The interaction between Prp43/DHX15 and Gno1/PinX1 is conserved across the evolution and is mediated by the G-patch domain [52].

Prp43 displays only weak helicase activity* in vitro* on DNA/RNA substrates with a single-stranded RNA tail, and G-patch proteins are able to stimulate this helicase activity [57]. This activity is strongly stimulated by Ntr1 and especially by the N-terminal truncation (1–122) that contains the G-patch domain, while mutants of conserved residues of the G-patch domain cannot stimulate the helicase activity [46]. Pfa1 is also shown to activate helicase activity of Prp43 through its C-terminal domain (574–767) containing the G-patch [53]. In humans, RBM5 activates helicase activity of DHX15 and mutations in the G-patch domain [51]. According to these results, G-patch proteins are able to stimulate the weak helicase activity of Prp43 and the G-patch domain of these proteins mediates this activation.

The G-patch partners of Prp43 also stimulate Prp43 ATPase activity. In human, RBM5 and GPATCH2 are able to stimulate ATPase activity of DHX15 [51, 54]. The C-terminal domain of Pfa1 (574–767) is sufficient to stimulate ATPase activity of Prp43. Interestingly, Pfa1 can stimulate ATPase activity with and without RNA, but optimal stimulation occurs in presence of RNA [53]. Fusions of Prp43 with different fragments of Ntr1 show that the G-patch domain (51–110) is directly responsible for the activation of Prp43 helicase and ATPase activity [58]. Similar results have been obtained for Gno1/PinX1. PinX1 is able to stimulate ATPase activity of Prp43 and mutations in the G-patch domain impede the activation [52].

**Figure 3:**
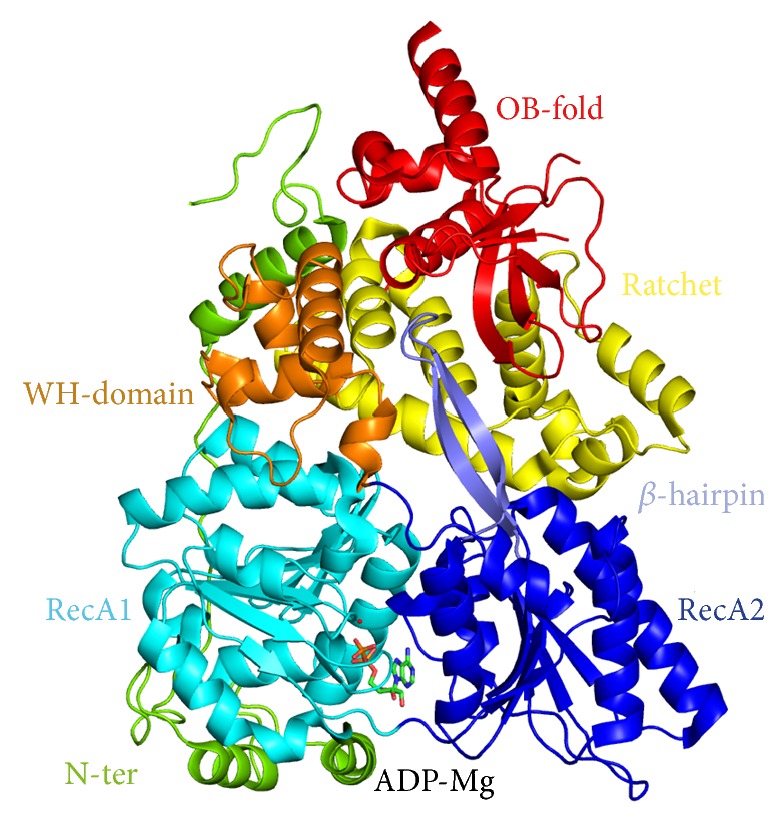
Structure of yeast Prp43 in complex with ADP (PDB code 2XAU). The protein contains six domains: the N-ter domain (green); the RecA1 and RecA2 domains which bind the nucleotide (cyan and dark blue, resp.); the WH domain (orange); the ratchet domain (yellow); and the OB-fold domain (red).

**Figure 4:**
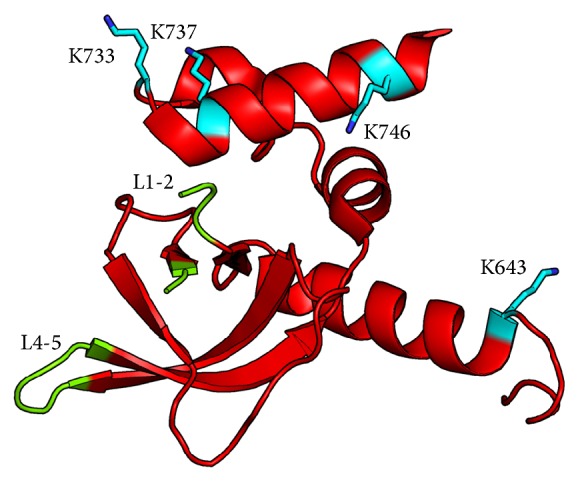
The OB-fold domain of Prp43 is implicated in the interaction of the helicase with Ntr1 and with RNA. The two loops L1-2 and L4-5 which have been involved in nucleic acid interaction [60] are colored in green. Residues that are involved in the interaction with Ntr1 according to cross-link experiments [58] are colored in cyan.

The structure of Prp43 has been solved by X-ray crystallography and this helicase contains six domains (Figure 3) including the two classical RecA-like domains and the OB-fold that is responsible for the interaction with RNA [59, 60]. Interestingly, Pfa1 fails to stimulate ATPase activity of the truncated version of Prp43 lacking the OB-fold domain. The Pfa1 C-terminal domain lacking the OB-fold domain does not interact with Prp43 [60]. Thus, the OB-fold domain of Prp43 seems to be a platform that mediates interaction with RNA and G-patch domain of its partners (Figure 4), probably by allosteric conformational rearrangements that would allow the enzyme to activate its ATPase activity.

Recently, Ficner and coworkers have investigated the role of the G-patch of Ntr1 in the interaction with Prp43 and with RNA. By circular dichroism, the G-patch domain is seen unstructured in solution, but it could form secondary structure elements during the interaction with Prp43 or nucleic acid. Cross-linking coupled to mass spectrometry shows that the interaction is mediated by the C-terminal domains of Prp43 and the N-terminal domain of Ntr1, including a residue in the G-patch domain. Ntr1 and Prp43 both interact with RNA. Moreover, binding of Ntr1 to Prp43 promotes structural rearrangements of Prp43, especially in the OB-fold, supporting a model of cooperative binding to RNA by Prp43 and Ntr1 [58] (Figure 4).

In agreement with the* in vitro* activation of the ATPase and helicase activities, the G-patch of DEAH/RHA partners seems to be directly responsible for their activation* in vivo*. Mutations in the G-patch of Spp2 impede the action of Prp2 in the spliceosome. The OB-fold domain of Prp2 is able to interact with Spp2 and mutations in this domain impede this interaction. Mutation of a residue of the G-patch of Spp2 can restore the interaction of Spp2 with the mutant of Prp2, indicating that the G-patch of Spp2 is implicated in the interaction with the C-terminal domain of Prp2 [43]. Therefore, the OB-fold of Prp2 interacts with the G-patch domain of Spp2 and the G-patch of Spp2 activates Prp2 function* in vivo*. Pfa1 (201–767) is sufficient to diminish accumulation of 20S pre-rRNA [53]. Truncation of the G-patch domain of Pfa1 is unable to complement growth defect of cells lacking Pfa1 and Ltv1. In the case of Pfa1, the G-patch domain also seems to be responsible for the activation of Prp43* in vivo*.

*Conclusions and Perspectives.* G-patch proteins are activators of DEAH/RHA helicases and the structural features of this activation are starting to be understood even if the overall mechanism remains elusive. Interestingly, not all the DEAH/RHA helicases are associated with a G-patch protein, and unidentified G-patch protein partners or additional G-patch proteins might be uncovered in metazoans. This possibility is supported by the fact that DHX15 is copurified with the ZIP-NuRD complex [39]. The lack of atomic structure of G-patch protein or DEAH/RHA helicase in complex with a G-patch protein is a limiting element to understand the precise structural features of the activation. These structures coupled to a better understanding of the activation of DEAH/RHA helicases could lead to an accurate model of this regulation. This model will be the key to understand how helicases can contribute to the fine regulation of essential processes in RNA metabolism. Comprehension of these mechanisms could help to understand how the interaction between G-patch protein and DEAH/RHA helicases can be implied in cancer as it has been demonstrated for DHX15 and GPATCH2 [54].

## Figures and Tables

**Table 1:** Summary of biological functions of DEAH/RHA helicases activated by a G-patch protein. The different biological processes, in which Prp43/DHX15 and Prp2/DHX16 and their activators are implied, are listed. The yeast orthologs are listed in parenthesis.

DEAH/RHA	Functions	Protein partner
DHX15 (Prp43)	Splicing	**TFIP11 (Ntr1); RBM5**
	Ribosome biogenesis	**SQS1 (Pfa1);** **PINX1 (Gno1)**
	**?**	**GPATCH2**
	Immunity	**?**

DHX16 (Prp2)	Splicing	**(Spp2)**

**Table 2:** Human DEAH/RHA helicases with no known G-patch activator and biological processes in which they are implied. For each protein, the identified yeast ortholog is listed in parenthesis.

DEAH/RHA	Functions
DHX38 (Prp16)	Splicing
DHX8 (Prp22)	Splicing
DHX37 (Dhr1)	Ribosome biogenesis
DHX32 (Dhr2)	Ribosome biogenesis
DHX29 (YLR419w)	Translation initiation; immunity
DHX33	Transcription; immunity
DHX9	Genomic stability; immunity; RNA interference
DHX29	Translation initiation; immunity
DHX35	Splicing
DHX34	mRNA decay
